# Restriction Spectrum Imaging As a Potential Measure of Cortical Neurite Density in Autism

**DOI:** 10.3389/fnins.2016.00610

**Published:** 2017-01-18

**Authors:** Ruth A. Carper, Jeffrey M. Treiber, Nathan S. White, Jiwandeep S. Kohli, Ralph-Axel Müller

**Affiliations:** ^1^Brain Development Imaging Laboratory, Department of Psychology, San Diego State UniversitySan Diego, CA, USA; ^2^School of Medicine, University of California San DiegoLa Jolla, CA, USA; ^3^Multimodal Imaging Laboratory, Department of Radiology, University of California San DiegoLa Jolla, CA, USA

**Keywords:** autism, diffusion, MRI, cerebral cortex, neurite, DTI, gray matter, connectivity

## Abstract

Autism postmortem studies have shown various cytoarchitectural anomalies in cortical and limbic areas including increased cell packing density, laminar disorganization, and narrowed minicolumns. However, there is little evidence on dendritic and axonal organization in ASD. Recent imaging techniques have the potential for non-invasive, *in vivo* studies of small-scale structure in the human brain, including gray matter. Here, Restriction Spectrum Imaging (RSI), a multi-shell diffusion-weighted imaging technique, was used to examine gray matter microstructure in 24 children with ASD (5 female) and 20 matched typically developing (TD) participants (2 female), ages 7–17 years. RSI extends the spherical deconvolution model to multiple length scales to characterize neurite density (ND) and organization. Measures were examined in 48 cortical regions of interest per hemisphere. To our knowledge, this is the first time that a multi-compartmental diffusion model has been applied to cortical gray matter in ASD. The ND measure detected robust age effects showing a significant positive relationship to age in all lobes except left temporal when groups were combined. Results were also suggestive of group differences (ASD<TD) in anterior cingulate, right superior temporal lobe and much of the parietal lobes, but these fell short of statistical significance. For MD, significant group differences (ASD>TD) in bilateral parietal regions as well as widespread age effects were detected. Our findings support the value of multi-shell diffusion imaging for assays of cortical gray matter. This approach has the potential to add to postmortem literature, examining intracortical organization, intracortical axonal content, myelination, or caliber. Robust age effects further support the validity of the ND metric for *in vivo* examination of gray matter microstructure in ASD and across development. While diffusion MRI does not approach the precision of histological studies, *in vivo* imaging measures of microstructure can complement postmortem studies, by allowing access to large sample sizes, a whole-brain field of view, longitudinal designs, and combination with behavioral and functional assays. This makes multi-shell diffusion imaging a promising technique for understanding the underlying cytoarchitecture of the disorder.

## Introduction

By general consensus, autism spectrum disorder (ASD) is a neurobiological disorder, likely of complex genetic, epigenetic, and possibly environmental origin, with brain development deviating from the typical path beginning in the prenatal period (Bailey et al., 1998; Palmen et al., 2004; Bauman and Kemper, 2005; Hutsler and and Casanova, 2016). Post-mortem studies are indispensible to our understanding of the underlying cellular anomalies. A reduction in the number of cerebellar Purkinje cells was among the earliest histologic reports (Bauman and Kemper, 1985), and a report of patchy neocortical thickening and laminar disorganization followed (Bailey et al., 1998). Increased cell packing density has been found in anterior cingulate, hippocampus and amygdala (Kemper and Bauman, 1998; Schumann and Amaral, 2006), and in prefrontal cortex by some (Bailey et al., 1998; Courchesne et al., 2011), but not all (Bauman and Kemper, 2005) research groups, while decreased density was found in fusiform gyrus (van Kooten et al., 2008). Increases in cortical cell packing density may relate to narrowed mini-columns in dorsolateral prefrontal cortex and superior temporal gyrus (Casanova et al., 2002, 2006; Buxhoeveden et al., 2006), suggesting a reduction in the amount of neuropil space surrounding neurons, which may reflect a decrease in inhibitory neurites in the affected regions. Ectopias (Bailey et al., 1998; Wegiel et al., 2010) and increased dendritic spine densities (Hutsler and Zhang, 2010) have also been reported. These neurostructural findings suggest altered rates of neurogenesis, delayed or reduced apoptosis or pruning or local failures of migration.

The postmortem literature is also quite variable, however, due to limitations which may be addressed through MRI and other *in vivo* imaging techniques. Some postmortem neuropathology is described as “patchy” at the individual case level (Bailey et al., 1998; Hutsler et al., 2007), and some findings are inconsistent across research groups or methods (Bailey et al., 1998; Bauman and Kemper, 2005; Hutsler et al., 2007). Furthermore, it is quite likely that histological differences that can be detected vary with the age of the case, particularly over the course of childhood, as indicated by *in vivo* MRI studies (Courchesne et al., 2001; Carper et al., 2002; Schumann et al., 2010; Hazlett et al., 2011). Such variability is difficult to overcome in postmortem studies, which are usually limited to small samples (averaging about 5 cases per study; Schumann and Nordahl, 2011) across wide age spans, with most cases in the adolescent or adult range. In addition, since histologic studies are extremely time consuming, studies are often limited to samples of only a few anatomical regions rather than whole-brain surveys. Longitudinal studies are furthermore impossible, limiting any developmental interpretations of post-mortem findings. *In vivo* imaging techniques may overcome many of these issues, allowing large sample sizes, whole-brain assessment, and longitudinal studies. However, examination of sub-voxel features such as dendritic or axonal organization and cortical cytoarchitecture has remained beyond the reach of *in vivo* imaging studies on ASD published to date.

The ability to describe cytoarchitecture and neuronal connectivity at the sub-millimeter level in living subjects would be a tremendous boon to research of neurodevelopmental and other neurologic disorders. Continuing improvements in system hardware and advances in acquisition techniques and sequence programming continue to push back the limits on spatial resolution. At the same time, new models for analysis of diffusion MRI allow examination of separate compartments within a single voxel. The combination of multi-shell diffusion acquisitions and multi-compartmental analysis approaches permits estimates of neurite (both axon and dendrite) content and organization within clinically manageable acquisition times (Jensen et al., 2005; Lu et al., 2006; Zhang et al., 2012; White et al., 2013). *Multi-shell* diffusion imaging (i.e., acquisition at multiple *b*-values and multiple diffusion directions), allows classification of diffusion at multiple length scales, disambiguating restricted (slow) diffusion from hindered (fast) diffusion. Inclusion of high *b*-values allows insight into micro-scale structures such as the organization and density of dendritic and axonal processes (neurites; Barazany et al., 2009; Raffelt et al., 2012; Assaf et al., 2013; Dell'Acqua et al., 2013). Here we used Restriction Spectrum Imaging (RSI, White et al., 2013), one such analysis approach, for *in vivo* examination of neurite organization within cerebral cortex in an ASD population.

RSI extends the spherical deconvolution model (Tournier et al., 2004) across these multiple length scales to characterize neurite density and organization at each imaged voxel. Analogous models have been used to examine white matter in one study of young adults with ASD (Lazar et al., 2014) but, to our knowledge, this is the first time that a multi-compartmental model has been applied to cortical gray matter in this population and the first time this age group has been addressed. We examined 48 cortical regions of interest per hemisphere in a population of 24 ASD and 20 TD children and adolescents.

## Materials and methods

### Participants

Participants ranged between 7 and 17 years of age and included both males and females. All potential ASD participants were administered the Autism Diagnostic Observation Schedule (ADOS, Lord et al., 2001), and their parents completed the Autism Diagnostic Interview-Revised (ADI-R, Rutter et al., 1995). Final diagnosis of Autism Spectrum Disorder was determined by a trained clinical psychologist according to DSM-V criteria (American Psychiatric Association, 2013) and with reference to ADOS and ADI-R scores. Children with known history of neurological disorders other than ASD (e.g., Fragile X syndrome, epilepsy) were excluded. Typically developing (TD) participants were recruited from the community, excluding anyone with a personal or family history of autism or a personal history of other neurologic or psychiatric conditions. Participants were also administered the Wechsler Abbreviated Scale of Intelligence (WASI, Wechsler, 1999), the Social Responsiveness Scale (SRS, Constantino and Gruber, 2005), and the Edinburgh Handedness Inventory (Oldfield, 1971). The study was approved by the University of California, San Diego, and San Diego State University Institutional Review Boards, with written informed consent and assent provided by all participants and caregivers.

### MRI data acquisition and preprocessing

MRI data were collected on a GE Discovery MR 750 3.0T system using an 8-channel head coil. Diffusion was measured with a multi-shell EPI sequence encoded for 45 non-collinear diffusion directions, (15 unique directions at each of 3 *b*-values: 500, 1500, and 4000 s/mm^2^) and 2 at b = 0 s/mm^2^ (in-plane resolution = 1.875 × 1.875 mm, thickness = 2.5 mm, TR = 7 s, TE = 87.4 ms, flip = 90°). An anatomical T1-weighted fast spoiled gradient echo (FSPGR) scan (1 mm^3^, TR = 8.108 s, TE = 3.172 ms, flip = 8°) was also acquired. Preprocessing of diffusion data was performed using in-house software and included eddy current correction (Zhuang et al., 2006), rigid body correction for motion with corresponding adjustments to the vector matrix, correction of susceptibility-induced field distortions (Holland et al., 2010), and correction for gradient non-linearities (Jovicich et al., 2006).

### Quality assessment and motion quantification

Multi-shell diffusion images were initially collected from 33 ASD and 24 TD children and adolescents. Average translation and rotation between acquisitions was calculated for each participant and considered for group matching. All image data, including each diffusion direction and *b*-value, were also visually inspected for motion-related signal dropout and other artifacts. The high *b*-value shell is particularly sensitive to motion-related dropout leading to a high exclusion rate. Seven subjects were excluded for excessive dropout (5 ASD, 2 TD) and an additional six for translation >1 mm or rotation >.01 radians (0.6 degrees, 4 ASD, 2 TD). Participants who were excluded did not differ significantly from those who were included with regard to age, IQ, or symptom severity (ADOS, ADI, SRS), for either subject group.

### Restriction spectrum imaging

The RSI model is based on the compartmentalization of water in brain tissue. Diffusion of water molecules within brain tissue is constrained by cell membranes and other structures and thus ranges from *restricted diffusion*, as in intra-cellular spaces where water is (on time scales examined here) unable to diffuse beyond the cellular or axonal membrane, to *free diffusion*, found in fluid spaces where diffusion is unencumbered by barriers such as membranes or large proteins (Le Bihan, 2012). Between these extremes, diffusion is *hindered*, e.g., in extracellular spaces where water must follow a tortuous path to pass around cell membranes or other obstacles, but is not enclosed by such barriers (Assaf and Basser, 2005). RSI applies a mathematical model (White et al., 2013, 2014) to determine the proportion of a voxel (*volume fraction*) and signal (*signal fraction*) stemming from hindered, restricted, or free water compartments and the geometry of diffusion within each of these compartments (isotropic or anisotropic). The algorithm is described in detail in the original validation study (White et al., 2013) and represents an extension of the linear spherical deconvolution model (Tournier et al., 2004; Dell'Acqua et al., 2007; Jian and Vemuri, 2007; Kaden et al., 2007) to multiple diffusion length scales. In the current application we used five diffusion length scales. The volume fraction of anisotropic restricted diffusion (the shortest length scale examined) is believed to reflect the relative density of neuronal processes (neurite density, ND; White et al., 2013). ND was calculated for each voxel, as were the fractional anisotropy (FA) and mean diffusivity (MD) derived from the diffusion tensor. The ND volume fraction was standardized to a 1–1000 range; FA ranged 0–1.

### Analysis of anatomical images and extraction of ROIs

Preprocessing of anatomical T1 scans included correction for gradient non-linearities (Jovicich et al., 2006) and brain extraction (Smith, 2002). A gray matter mask was derived for each subject (Avants et al., 2011b) and affine registration was used to align each participant's T1 to the corresponding RSI image and to a sample-specific template in MNI space which had been derived using Advanced Normalization Tools (ANTS, Avants et al., 2010, 2011a). This allowed backward transformation of the Harvard-Oxford cortical atlas (http://fsl.fmrib.ox.ac.uk/fsl/fslwiki/Atlases) from MNI space to each individual's native diffusion space, providing 48 gyral-level ROIs for each hemisphere. Average ND, FA, and MD within gray matter were calculated for each of these ROIs and for the overall cerebral lobes (see Supplementary Table 1 for ROIs and their lobar designations).

## Results

The final sample included 24 ASD participants (5 female) aged 7–17 years, and 20 TD participants (2 female) aged 8–17 years. Groups were well matched for age, non-verbal IQ, and motion measures with all *p* > 0.5 (Table 1). The ASD group had lower verbal IQ as is frequently found in this socio-communicative disorder.

**Table 1:** **Demographics**.

	**ASD (*n* = 24) mean ± *SD* [range]**	**TD (*n* = 20) mean ± *SD* [range]**	***p*****-value**
Age (years)	13.41 ± 3.30	13.72 ± 2.91	0.693
	[7.43–17.98]	[8.19–17.69]	
WASI_VIQ	91.83 ± 17.25	104.45 ± 10.28	0.005
	[56–118]	[73–126]	
WASI_NVIQ	98.54 ± 19.33	101.65 ± 15.06	0.552
	[53–140]	[62–123]	
Avg. Translation	0.61 ± 0.11	0.62 ± 0.12	0.791
	[0.41–0.86]	[0.33–0.85]	
Avg. Rotation	0.003 ± 0.0021	0.0029 ± 0.0025	0.893
	[0.0012–0.0081]	[0.0011–0.0098]	
SRS Total	84.13 ± 8.53	43.15 ± 5.61	<0.001
	[62–100]	[35–52]	
ADOS-2 SA	12.26 ± 3.86		
	[6–20]		
ADOS-2 RRB	3.44 ± 2.33		
	[1–12]		
ADOS-2 Severity	8.32 ± 1.70		
	[4–10]		
ADI Soc	18.50 ± 4.08		
	[13–28]		
ADI Comm	13.25 ± 4.40		
	[6–24]		
ADI Rep	5.96 ± 2.48		
	[1–12]		
Female	*n* = 5	*n* = 2	
Left Handed	*n* = 5	*n* = 2	

*ASD, Autism Spectrum Disorder; TD, Typically Developing; WASI, Wechsler Abbreviated Scales of Intelligence; VIQ, Verbal IQ; NVIQ, Non-verbal IQ; SRS, Social Responsiveness Scale; ADOS-2, Autism Diagnostic Observation Schedule-2nd edition; SA, Social Affect; RRB, Restricted and Repetitive Behavior; ADI-R, Autism Diagnostic Interview, Revised; Soc, Social interaction subscale; Comm, Communication subscale; Rep, Restricted and Repetitive Behaviors subscale*.

Linear regressions were performed on ND, FA, and MD measures separately for each lobe and hemisphere with age, group, group-by-age interaction, and a constant included in each model. The false discovery rate (FDR, Benjamini and Hochberg, 1995) was used to correct for multiple comparisons. All regressions performed on a single dependent variable (ND, FA, or MD) were included within a statistical family with the significance of each overall *F*-test included in that correction. Coefficients were corrected in a similar fashion.

### Lobar effects

**Figure 1:**
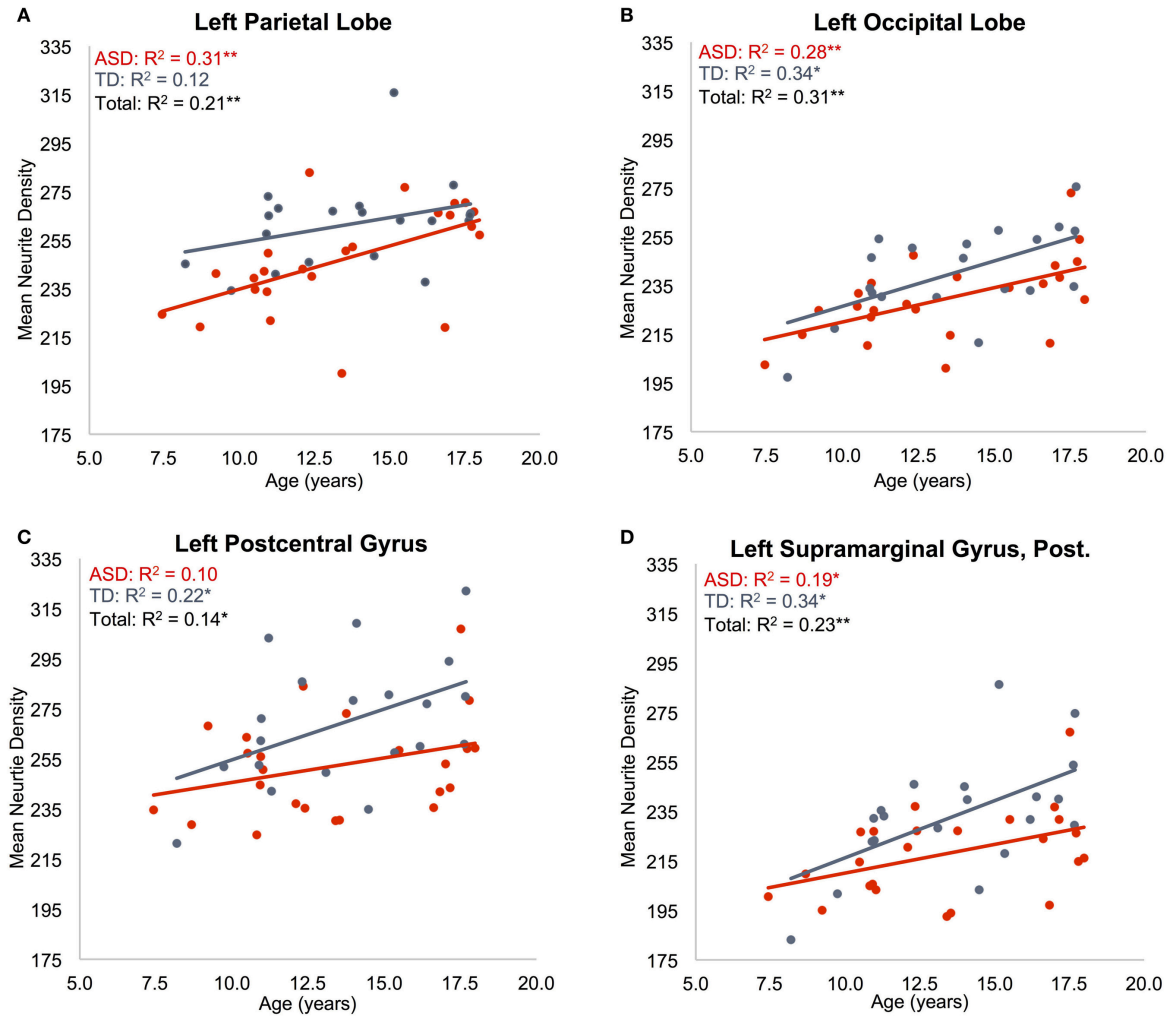
**Effects of age on neurite density**. Mean ND is shown as a function of subject age for: **(A)** left parietal lobe, **(B)** left occipital lobe, **(C)** left postcentral gyrus, **(D)** posterior division of left supramarginal gyrus. ASD indicated in red, TD indicated in blue. Neurite density standardized to a 0–1000 range. ^*^p < 0.05, ^**^p < 0.005.

**Figure 2:**
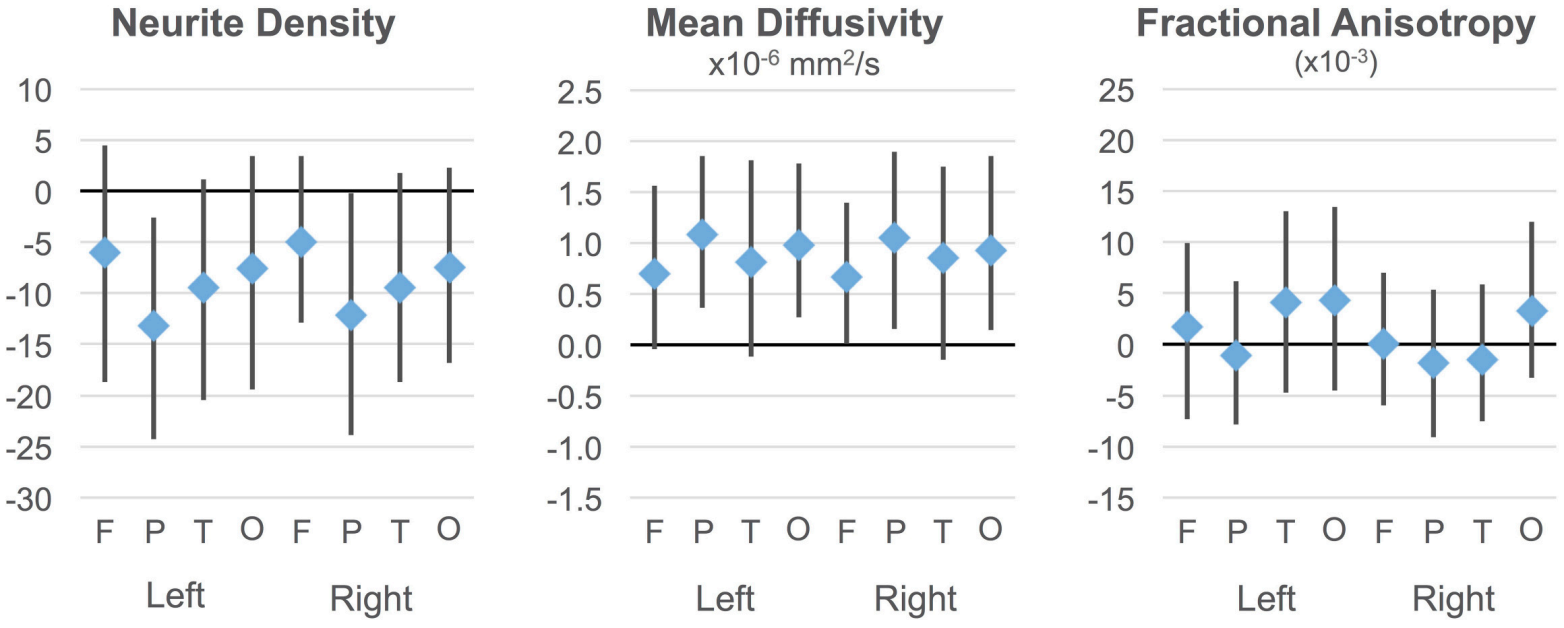
**Group differences in ND, MD, and FA**. Group differences and 95% confidence intervals are shown for each cortical lobe. Positive values indicate ASD >TD. Neurite density standardized to a 0–1000 range.

Regressions were significant for ND in all lobes in the right hemisphere and for frontal, parietal, and occipital lobes in the left hemisphere (Table 2). The effects of age were significant and positive (increasing with age) in all of these. Rates ranged from 1.38 per year (left occipital lobe) to 4.11 per year (right frontal lobe) with the volume fraction standardized to a 1–1000 scale (Figures 1A,B). Group differences showed reduced ND in left parietal and left occipital lobes in ASD compared to TD participants (Figure 2) with moderate effect sizes ranging from 0.37 to 0.75, but these did not survive correction for multiple comparisons. Interactions between group and age were not significant.

**Table 2:** **Linear regression results (***p***-values) for neurite density, fractional anisotropy, and mean diffusivity by lobe**.

**Lobe**			**Group**	**Age**	**Age X Group**	**Overall *F*-test**
ND	Left	Frontal	0.290	0.003^*^	0.425	0.011^*^
		Parietal	0.018	0.002^*^	0.471	0.001^*^
		Temporal	0.215	0.197	0.622	0.270
		Occipital	0.043	<0.001^*^	0.657	<0.001^*^
	Right	Frontal	0.237	<0.001^*^	0.281	<0.001^*^
		Parietal	0.051	0.015^*^	0.397	0.011^*^
		Temporal	0.106	0.005^*^	0.995	0.013^*^
		Occipital	0.121	0.003^*^	0.269	0.004^*^
FA	Left	Frontal	0.678	0.807	0.275	0.709
		Parietal	0.751	0.418	0.045	0.147
		Temporal	0.393	0.748	0.159	0.470
		Occipital	0.312	0.659	0.663	0.726
	Right	Frontal	0.984	0.090	0.417	0.284
		Parietal	0.640	0.083	0.035	0.037
		Temporal	0.656	0.147	0.698	0.438
		Occipital	0.368	0.561	0.453	0.664
MD	Left	Frontal	0.091	0.001^*^	0.788	0.002^*^
		Parietal	0.011	<0.001^*^	0.760	<0.001^*^
		Temporal	0.115	0.091	0.665	0.137
		Occipital	0.027	<0.001^*^	0.840	<0.001^*^
	Right	Frontal	0.080	<0.001^*^	0.547	<0.001^*^
		Parietal	0.020	<0.001^*^	0.864	<0.001^*^
		Temporal	0.088	0.025^*^	0.509	0.044
		Occipital	0.041	<0.001^*^	0.601	<0.001^*^

*Uncorrected p-values of each coefficient shown*.

* *Significant following FDR correction for multiple comparisons. Tests of each coefficient and dependent variable (ND, FA, MD) treated as a statistical family for FDR*.

On the MD measure, regressions were significant bilaterally in frontal, parietal and occipital lobes, but not temporal lobes. All of these showed significant negative effects of age (decreasing with age) with rates ranging from −2.445 × 10^−6^ mm^2^/s per year (left frontal lobe) to −3.875 × 10^−6^ mm^2^/s per year (left parietal lobe). MD was higher in ASD in the parietal and occipital lobes bilaterally (Figure 2) again with medium effect sizes (0.49 to 0.67). However, these did not survive correction for multiple comparisons. There were no significant interactions. By contrast, similar linear regression analyses for FA values did not reach significance after correction for multiple comparisons.

### Localized effects

Individual ROIs were also examined for a more localized understanding of effects in MD and ND (see Supplementary Table 1 for list of ROIs examined), using similar linear regression models. For each dependent variable, all regions were included bilaterally to correct each coefficient for multiple comparisons (48 regions x 2 hemispheres).

Regressions on ND were significant for all regions on the dorsolateral aspect of the right frontal lobe as well as the right anterior cingulate, paracingulate, operculum and insula (Supplementary Table 2, Figures 1C,D). Left frontal effects were restricted to anterior cingulate, paracingulate, central operculum, and posterior portions of the dorsal surface. Regressions were also significant for all subregions of parietal lobes (except bilateral superior parietal lobule, right precuneus, and right postcentral), a few bilateral temporal regions including posterolateral regions, planum temporale, Heschl's gyrus, and right temporal occipital fusiform gyrus, and portions of occipital lobe including bilateral posterior cingulate. All of these except left Heschl's gyrus exhibited significantly greater ND with increasing age.

Group effects (see Supplementary Figure 1 for group differences and confidence intervals) suggested that ND was reduced in ASD in a number of regions (e.g., anterior cingulate, precentral and supramarginal gyri bilaterally, parts of superior temporal gyrus, fusiform, and planum temporale on the right), but only left temporal occipital fusiform cortex survived correction for multiple comparisons (and overall regression was not significant). The only significant group-by-age interaction was in right anterior cingulate with ND increasing more rapidly with age in ASD.

With only one exception (right temporal occipital fusiform), all regions that showed significant positive age effects on ND also showed significant negative effects on the MD measure (Supplementary Figure 2, Supplementary Table 3). In addition, left superior parietal lobule was affected, and more of left frontal lobes (left middle frontal, subcallosal gyri), and occipital lobes (bilateral intracalcarine cortex and pole, right lateral occipital gyrus, left supracalcarine, cuneal, and lingual regions) showed age effects.

Group differences on MD did not survive correction, but the tendency was toward higher MD in ASD than TD particularly in parietal regions and in several frontal and occipital regions (see Supplementary Figure 2 for group differences and confidence intervals). Interactions were not significant.

## Discussion

The present study is—to our knowledge—the first to apply a multi-compartmental diffusion model using multishell MRI to the study of *cortical gray matter* microstructure in ASD. We found robust age effects for both ND (increasing) and MD (decreasing), but not FA, a measure more suited to tissues with well-aligned microstructure such as in the deep white matter. Trends toward decreased ND and increased MD in ASD did not survive correction for multiple comparisons when examined at the lobar level. More localized examination again showed robust age effects in the ND and MD measures, and a significant group difference was found on ND (ASD<TD) in the left temporal occipital fusiform gyrus. While other ND differences did not survive correction for multiple comparisons, examination of confidence intervals (Supplementary Figures 1, 2) suggests that larger sample sizes or improvements in signal-to-noise and motion control (see Limitations) may support such differences.

Our findings indicate that multi-shell, multi-compartmental approaches may provide a valuable addition to our ability to examine gray matter microstructure in ASD and other disorders. While the RSI derived ND measure may not be as sensitive as the tensor-derived MD measure, it offers greater interpretability and specificity as discussed below.

### Neurite density and mean diffusivity

The ND measure is likely driven primarily by axons, and particularly myelinated axons, rather than dendrites within cerebral cortex. At the diffusion time-scales examined here, unmyelinated neurites allow some amount of water exchange across the cellular membrane. Since the ND measure is derived from elements with cylindrical symmetry, wherein diffusion is restricted in the direction transverse to the cylinder, but relatively unencumbered along the long axis, unmyelinated elements will contribute less overall signal to the neurite water compartment due to greater water exchange. Hence, lower ND such as that seen in younger participants likely reflects: (1) lower density of myelinated axons, (2) thinner myelin (allowing greater average water exchange), or possibly (3) smaller average caliber of myelinated axons which would be associated with less water in the restricted cylindrical pool.

Cortical MD may be driven by the same factors (but in the opposite direction) since ND and MD measures will tend to correlate inversely with each other. MD is highest where diffusion is free and lowest where it is restricted (e.g., areas of high ND). However, MD is derived from a tensor model and does not distinguish intracellular from extracellular compartments as do RSI derived measures. Other possible causes of MD effects therefore cannot be excluded in regions where ND effects were not detected. Inflammatory responses also lead to increased MD due to increased tissue water (Alexander et al., 2007) but would not be expected to alter intracellular measures such as ND. Signs of inflammation have been reported in ASD (Vargas et al., 2005; Zimmerman et al., 2005; Morgan et al., 2010; Suzuki et al., 2013) so this potential contributor must be considered. Alternatively, MD may simply be a more robust measure than ND when examining gray matter. With only about 25% of the cortical diffusion volume fraction being restricted, ND signal-to-noise will be lower than in the composite MD measure. The relative simplicity of the tensor model may also make measures such as MD more robust than those derived from the more complex RSI model. However, since effect sizes for ND ranged from medium to large (0.37 to 0.75) the lack of more significant findings may have been due to limited sample size, accompanied by expected variability due to the known etiological heterogeneity in ASD (Geschwind and State, 2015).

### Age effects

The validity of the ND metric was supported by robust maturational effects detected in our study. ND increased significantly with age in all lobes except left temporal when groups were combined, showing clear sensitivity to developmental change. At first glance, the direction of change may appear unexpected: The number of cortical synapses, and presumably the complexity of dendritic branches, begins to decrease prior to age 8 years (Huttenlocher and Dabholkar, 1997), while cortical gray matter volume stabilizes (Courchesne et al., 2000), so that a measure of “neurite density” might be expected to decline during this period. However, as described above, the ND measure is probably particularly sensitive to the degree of axonal myelination, which continues well into adulthood. This is supported by multi-shell diffusion studies on neurotypical white matter development that also found age related increases in intraneurite compartments using compartmental diffusion models other than RSI. In a recent abstract, Chang et al. (2015) reported increasing intra-axonal volume fractions across childhood and adolescence using neurite orientation dispersion and density imaging (NODDI). Significant age related increases were also reported by Jelescu et al. (2015) in infants and toddlers, and by Billiet et al. (2015) in adults, with both of these using both NODDI and diffusion kurtosis imaging. These latter studies concluded that the age effects must be at least partially reflective of ongoing myelination, rather than strictly of intra-axonal volume fractions. Indeed, the same NODDI measure was found to correlate with direct staining of myelin in a rat model (Jespersen et al., 2007).

### Localization of group trends

While group differences on ND were only marginal,—surviving correction for multiple comparisons in only a single region—and therefore must be viewed with substantial caution, the localization of these results deserves some consideration in context of the postmortem literature. ND tended to be lower in ASD than TD in bilateral anterior cingulate gyri, for which increased cell packing density has been reported in several ASD post-mortem cases (Kemper and Bauman, 1998; Schumann and Amaral, 2006). This region also showed the only group-by-age interaction surviving correction, with the ASD group showing a greater increase in ND with age than TD. One of these postmortem studies also examined axons just beneath the cortex (Zikopoulos and Barbas, 2010). In that study, axons were sampled from white matter beneath four areas of frontal lobe in five adult ASD postmortem cases, finding a relative shift from larger to smaller caliber axons exiting the anterior cingulate cortex. As discussed above, smaller myelinated axons are one potential source of ND reductions, which would be consistent with our findings. Zikopoulos and Barbas (2010) also found decreased myelin thickness in axons exiting orbital frontal cortex compared to controls. But, while myelin thickness is another potential source of ND changes, we found little evidence of a group difference in orbital cortex. Other areas where shifts in cell packing density have been reported in ASD include the hippocampus and amygdala, which were not examined here, the prefrontal cortex (though not consistently), and potentially the superior temporal gyrus given findings of narrowed minicolumns. In our sample, ND did tend to be lower in the ASD group in the superior temporal gyrus and its superior aspect (Heschl's gyrus, planum temporale) primarily in the right hemisphere. We found no evidence of prefrontal differences. One group has reported *decreased* cell density in fusiform cortex, whereas we found lower ND that was limited to the posterior aspect of the gyrus.

## Limitations

The absence of significant group differences in RSI may partly reflect the inter-case variability of cytoarchitectonic abnormalities in ASD, commonly found in the literature (Bailey et al., 1998; Hutsler et al., 2007; Wegiel et al., 2010). Additionally, some neuropathologies may not affect the same regions of cortex across all ASD cases, such as laminar differences that have been described as “patchy” (Hutsler et al., 2007). Group-wise analyses such as those used here would not be sensitive to such subject-specific anomalies. Notably, one recent study demonstrated the utility of a multi-compartment diffusion approach for identification of focal cortical dysplasias, not unlike those reported in ASD cases, on a case-wise basis (Winston et al., 2014). Intracellular volume fraction, their marker of neurite density, highlighted focal dysplasias more prominently than traditional diffusion or structural imaging. With a large normative sample, it might be possible to detect focal dysplasias *in vivo* on a case-wise basis in ASD and other disorders using RSI or other compartmental diffusion models.

As with all MRI methodologies, subject motion during scanning can be an issue, particularly when comparing groups that may differ in their likelihood of motion. We thoroughly screened all scans for subject motion resulting in well-matched subject groups. However, better protection of multi-shell diffusion sequences from motion may be possible. Our highest *b*-value shell (b = 4000 s/mm^2^) was particularly susceptible to motion-related slice dropout, forcing us to exclude c. 27% of ASD participants. This could be improved either by reducing the maximum gradient strength while losing sensitivity to the shortest diffusion distances or, more simply, by repeating the acquisition of this highest shell to allow signal averaging.

Although RSI provides insight to subvoxel neural content in the form of separate intra and extracellular compartments, partial volume effects are still relevant. Voxel size was large (8.8 mm^3^) leading to inclusion of both gray and white matter within voxels and averaging across lamina. Higher resolutions may be possible with accelerated imaging techniques, but are unlikely to reach sublaminar resolution in the near future.

RSI is not sensitive to all types of cytoarchitectonic anomalies reported in the ASD literature, such as dysmorphology of specific cell types, ectopias, and abnormal dendritic spine density.

The sample examined here spanned a broad and developmentally complex age range, from 7 to 17 years. After controlling for subject motion, sample sizes of 24 and 20 participants per group were somewhat limited for such a broad range. We were also limited to participants who were relatively high functioning in order to maximize subject cooperation during scanning. Results derived here may not, therefore, generalize to lower-functioning ASD populations or to populations outside of the examined age range.

## Conclusion

DWI methods are typically restricted to examination of white matter. However, RSI shows substantial promise for microstructural examination of gray matter in ASD. The method is sensitive to effects of age, suggests that group differences may be detected with larger sample sizes, and offers greater interpretability than the traditional diffusion tensor measures FA and MD. The robust age effects that were found for ND support the validity and sensitivity of multi-shell, multi-compartment DWI for *in vivo* examination of gray matter in developmental populations. While RSI is not sensitive to all types of cytoarchitectonic anomalies reported in the ASD literature, measures are likely to reflect intracortical axonal content, myelination, and caliber, which have received limited attention in postmortem studies and can thus complement these in important ways. In the context of fundamental advantages of *in vivo* studies, which can be combined with functional (functional imaging or behavioral) assays, provide a whole brain field of view, and can be administered in longitudinal designs, the first findings reported here suggest that multishell diffusion imaging may be a promising complement to postmortem neurohistology in ASD.

## Author contributions

RC, NW, and RM contributed to the conception and design of the work; RC, JT, and JK contributed to the processing, analysis, and presentation of data; all authors contributed to the manuscript itself.

## Funding

This study was supported by National Institutes of Health grants R01-MH081023 and K01-MH097972, and by National Science Foundation Grant No. 1430082.

### Conflict of interest statement

The authors declare that the research was conducted in the absence of any commercial or financial relationships that could be construed as a potential conflict of interest.
