# Supplementary material for: Restriction Spectrum Imaging As a Potential Measure of Cortical Neurite Density in Autism
Source: Front Neurosci. 2017 Jan 18;10:610. doi: 10.3389/fnins.2016.00610 (PMC5241303; doi:10.3389/fnins.2016.00610)

Supplementary Material

Restriction Spectrum Imaging as a potential measure of cortical neurite density in autism

Ruth A. Carper[1]*, Jeffrey M. Treiber[2], Nathan S. White[3], Jiwandeep S. Kohli[1], Ralph-Axel Müller[1]

*** Correspondence:** Ruth A. Carper, Brain Development Imaging Laboratory, Department of Psychology, San Diego State University, San Diego, CA, USA.\

1.1 Supplementary Tables

(continues on next page)

Supplementary Table 1. Regions of Interest by Lobe. Regions examined in analyses, their abbreviations, and the lobes to which they were assigned. Regions were derived from the Harvard-Oxford cortical atlas http://www.cma.mgh.harvard.edu/).

**Frontal Lobe**		**Temporal Lobe**	
Frontal Pole	FrPole	Temporal Pole	TePole
Insular Cortex	Ins	Superior Temporal Gyrus, anterior division	STG-a
Superior Frontal Gyrus	SFG	Superior Temporal Gyrus, posterior division	STG-p
Middle Frontal Gyrus	MFG	Middle Temporal Gyrus, anterior division	MTG-a
Inferior Frontal Gyrus, pars triangularis	IFG-tri	Middle Temporal Gyrus, posterior division	MTG-p
Inferior Frontal Gyrus, pars opercularis	IFG-op	Middle Temporal Gyrus, temporooccipital part	MTG-to
Precentral Gyrus	PrCG	Inferior Temporal Gyrus, anterior division	ITG-a
Frontal Medial Cortex	FMed	Inferior Temporal Gyrus, posterior division	ITG-p
Juxtapositional Lobule Cortex (Suppl Motor Cortex)	SMC	Inferior Temporal Gyrus, temporooccipital part	ITG-to
Subcallosal Cortex	SubCalG	Parahippocampal Gyrus, anterior division	PaHipG-a
Paracingulate Gyrus	ParaCgG	Parahippocampal Gyrus, posterior division	PaHipG-p
Cingulate Gyrus, anterior division	CgG-a	Temporal Fusiform Cortex, anterior division	TFus-a
Frontal Orbital Cortex	FOrb	Temporal Fusiform Cortex, posterior division	TFus-p
Frontal Operculum Cortex	FOperc	Temporal Occipital Fusiform Cortex	TOccFus
Central Opercular Cortex	COperc	Planum Polare	PPol
		Heschl's Gyrus (includes H1 and H2)	HG
		Planum Temporale	PTemp
**Parietal Lobe**		**Occipital Lobe**	
Postcentral Gyrus	PoCG	Lateral Occipital Cortex, superior division	LatO-s
Superior Parietal Lobule	SupPL	Lateral Occipital Cortex, inferior division	LatO-i
Supramarginal Gyrus, anterior division	SuMarG-a	Intracalcarine Cortex	Intracalc
Supramarginal Gyrus, posterior division	SuMarG-p	Cingulate Gyrus, posterior division	CgG-p
Angular Gyrus	AngG	Cuneal Cortex	Cun
Precuneous Cortex	PrCun	Lingual Gyrus	LingG
Parietal Operculum Cortex	POperc	Occipital Fusiform Gyrus	OFusG
		Supracalcarine Cortex	SupCalc
		Occipital Pole	OcPole

Supplementary Table 2. Linear regression results (p-values) on Neurite Density by region

	Region	Left Hemisphere				Right Hemisphere			
		Group	Age	Age X Group	Overall F-test	Group	Age	Age X Group	Overall F-test
Frontal	Frontal Pole	0.71	0.91	0.50	0.87	0.98	0.38	0.32	0.59
	Insular Cortex	0.74	0.94	0.75	0.97	0.56	0.00	0.53	0.01
	Sup. Frontal G.	0.12	0.00	0.51	0.01	0.24	0.00	0.53	0.00
	Middle Frontal G.	0.24	0.04	0.72	0.10	0.34	0.00	0.38	0.00
	Inf. Frontal G., triangularis	0.53	0.61	0.84	0.87	0.53	0.00	0.22	0.00
	Inf. Frontal G., opercularis	0.87	0.55	0.97	0.94	0.67	0.00	0.12	0.00
	Precentral G.	0.05	0.00	0.32	0.00	0.03	0.00	0.06	0.00
	Frontal Medial Cortex	0.19	0.05	0.85	0.11	0.99	0.67	0.89	0.98
	Suppl. Motor/Juxtapositional	0.21	0.00	0.60	0.00	0.16	0.00	0.60	0.00
	Subcallosal Cortex	0.16	0.02	0.10	0.03	0.43	0.00	0.40	0.01
	Paracingulate G.	0.56	0.00	0.59	0.02	0.91	0.00	0.68	0.01
	Cingulate G., ant. div.	0.02	0.00	0.22	0.00	0.03	0.00	0.00	0.00
	Frontal Orbital Cortex	0.08	0.51	0.51	0.29	0.62	0.18	0.40	0.42
	Frontal Operculum Cortex	0.92	0.11	0.58	0.37	0.43	0.00	0.79	0.00
	Central Opercular Cortex	0.72	0.00	0.69	0.01	0.43	0.00	0.26	0.00
Parietal	Postcentral G.	0.02	0.01	0.91	0.01	0.17	0.02	0.33	0.03
	Sup. Parietal Lobule	0.04	0.19	0.57	0.08	0.30	0.86	0.54	0.64
	Supramarginal G., ant. div.	0.05	0.00	0.69	0.00	0.31	0.00	0.30	0.00
	Supramarginal G., post. div.	0.02	0.00	0.36	0.00	0.02	0.00	0.24	0.00
	Angular G.	0.13	0.00	0.65	0.00	0.03	0.01	0.26	0.00
	Precuneous Cortex	0.89	0.00	0.75	0.00	0.55	0.01	0.23	0.03
	Parietal Operculum Cortex	0.32	0.00	0.79	0.00	0.50	0.00	0.72	0.00
Temporal	Temporal Pole	0.58	0.59	0.45	0.77	0.29	0.03	0.51	0.10
	Sup. Temporal G., ant. div.	0.77	0.08	0.76	0.36	0.27	0.15	0.85	0.32
	Sup. Temporal G., post. div.	0.99	0.01	0.45	0.05	0.03	0.00	0.96	0.00
	Middle Temporal G., ant. div.	0.98	0.75	0.35	0.78	0.76	0.14	1.00	0.51
	Middle Temporal G., post. div.	0.63	0.25	0.29	0.37	0.60	0.01	0.69	0.06
	Middle Temporal G., temporooccip.	0.38	0.00	0.39	0.00	0.26	0.00	0.75	0.01
	Inf. Temporal G., ant. div.	0.93	0.25	0.58	0.67	0.52	0.37	0.63	0.70
	Inf. Temporal G., post. div.	0.39	0.29	0.04	0.12	0.89	0.00	0.49	0.03
	Inf. Temporal G., temporooccip.	0.42	0.00	0.81	0.01	0.96	0.20	0.12	0.28
	Parahippocampal G., ant. div.	0.70	0.99	0.76	0.97	0.92	0.11	0.22	0.28
	Parahippocampal G., post. div.	0.01	0.93	0.67	0.05	0.09	0.10	0.16	0.06
	Temporal Fusiform Cortex, ant. div.	0.35	0.88	0.05	0.14	0.83	0.10	0.53	0.39
	Temporal Fusiform Cortex, post.div.	0.13	0.38	0.60	0.28	0.57	0.55	0.78	0.83
	Temporal Occipital Fusiform Cortex	0.00	0.96	0.79	0.04	0.01	0.01	0.67	0.01
	Planum Polare	0.85	0.18	0.47	0.48	0.43	0.01	0.73	0.03
	Heschl's G. (includes H1 and H2)	0.02	0.03	0.60	0.01	0.01	0.00	0.26	0.00
	Planum Temporale	0.32	0.00	0.77	0.01	0.01	0.00	0.18	0.00
Occipital	Lateral Occipital Cortex, sup. div.	0.10	0.01	0.54	0.01	0.15	0.02	0.77	0.05
	Lateral Occipital Cortex, inf. div.	0.66	0.00	0.72	0.01	0.20	0.08	0.90	0.16
	Intracalcarine Cortex	0.97	0.00	0.97	0.03	0.12	0.03	0.73	0.06
	Cingulate G., post. div.	0.44	0.00	0.66	0.00	0.86	0.00	0.67	0.01
	Cuneal Cortex	0.39	0.03	0.62	0.13	0.21	0.08	0.88	0.18
	Lingual G.	0.01	0.28	0.69	0.04	0.02	0.69	0.68	0.09
	Occipital Fusiform G.	0.03	1.00	0.66	0.15	0.12	0.23	0.52	0.23
	Supracalcarine Cortex	0.57	0.04	0.88	0.19	0.02	0.00	0.23	0.00
	Occipital Pole	0.37	0.09	0.14	0.08	0.98	0.02	0.09	0.03

Uncorrected p-values of each coefficient shown. Shaded cells indicate values that were significant following FDR correction for multiple comparisons. Tests of each coefficient treated as a statistical family, combining across lobes and hemispheres, for FDR. Abbreviations: div.=division, G.=Gyrus, Suppl.=Supplementary, temporooccip.=temporooccipital, ant.=anterior, post.=posterior, inf.=inferior, sup.=superior.

Supplementary Figure 1. Group differences in ND. Group differences and 95% confidence intervals are shown for each region examined. Positive values indicate ASD>TD. Neurite density standardized to a 0-1000 range. Abbreviations as listed in Supplementary Table 1.

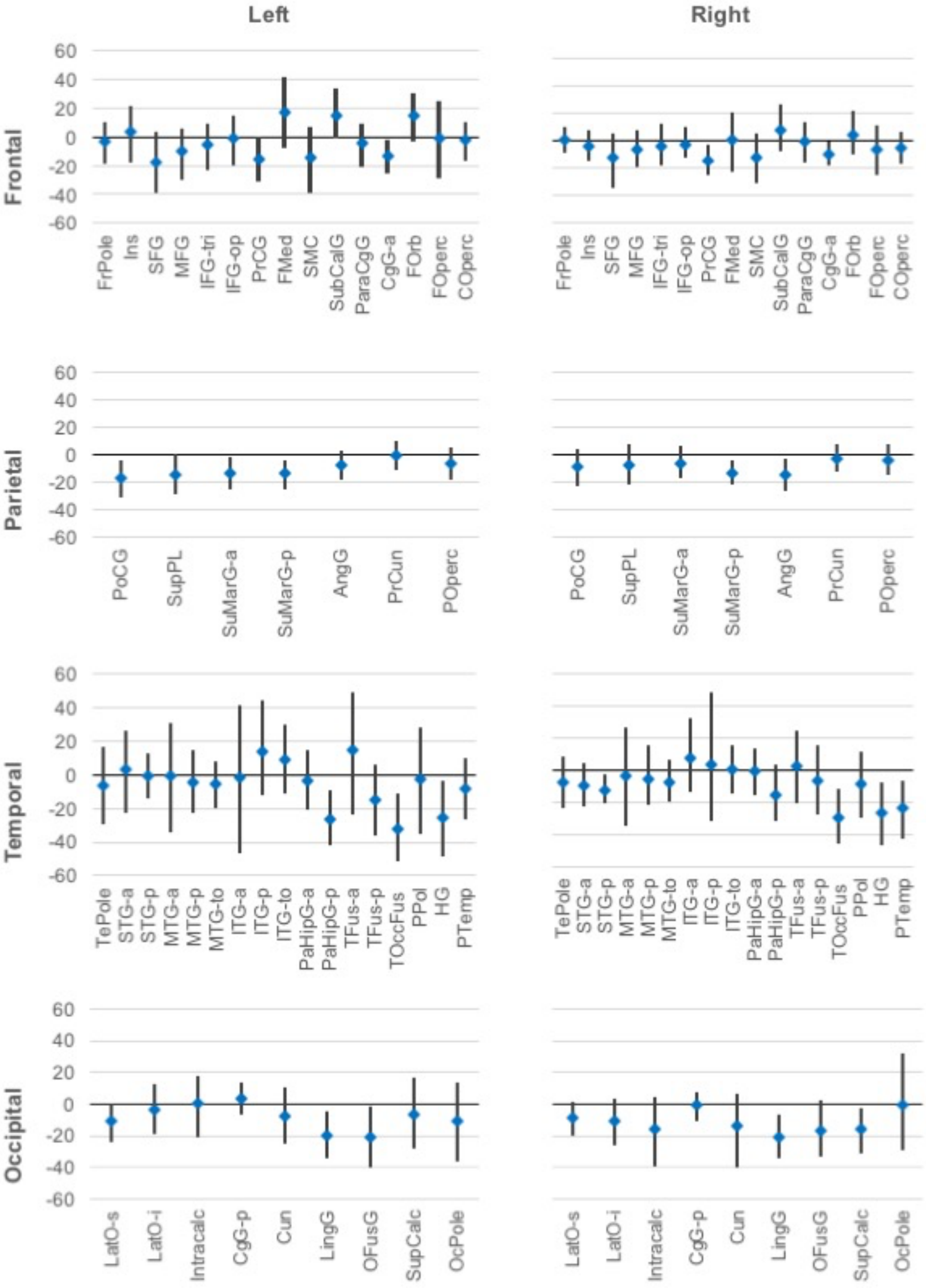

Supplementary Table 3. Linear regression results (p-values) on Mean Diffusivity by region

	Region	Left Hemisphere				Right Hemisphere			
		Group	Age	Age X Group	Overall F-test	Group	Age	Age X Group	Overall F-test
Frontal	Frontal Pole	0.40	0.47	0.97	0.72	0.29	0.08	0.53	0.16
	Insular Cortex	0.72	0.25	0.48	0.60	0.25	0.00	0.42	0.02
	Sup. Frontal G.	0.03	0.00	0.52	0.00	0.07	0.00	0.64	0.00
	Middle Frontal G.	0.05	0.01	0.79	0.01	0.06	0.00	0.69	0.00
	Inf. Frontal G., triangularis	0.08	0.18	0.74	0.17	0.13	0.00	0.69	0.00
	Inf. Frontal G., opercularis	0.32	0.03	0.95	0.12	0.21	0.00	0.33	0.00
	Precentral G.	0.01	0.00	0.24	0.00	0.01	0.00	0.06	0.00
	Frontal Medial Cortex	0.09	0.30	0.12	0.06	0.24	0.87	0.55	0.55
	Suppl. Motor Cortex/Juxtapositional	0.10	0.00	0.59	0.00	0.08	0.00	0.61	0.00
	Subcallosal Cortex	0.62	0.00	0.13	0.01	0.63	0.00	0.26	0.00
	Paracingulate G.	0.42	0.00	0.91	0.01	0.80	0.00	0.41	0.00
	Cingulate G., ant. div.	0.01	0.00	0.03	0.00	0.03	0.00	0.00	0.00
	Frontal Orbital Cortex	0.94	0.22	0.75	0.63	0.82	0.07	0.61	0.27
	Frontal Operculum Cortex	0.62	0.01	0.89	0.09	0.44	0.00	0.67	0.00
	Central Opercular Cortex	0.47	0.00	0.96	0.02	0.23	0.00	0.74	0.00
Parietal	Postcentral G.	0.02	0.00	0.65	0.00	0.09	0.00	0.31	0.00
	Sup. Parietal Lobule	0.01	0.01	0.60	0.00	0.02	0.12	0.39	0.03
	Supramarginal G., ant. div.	0.04	0.00	0.58	0.00	0.19	0.00	0.66	0.00
	Supramarginal G., post. div.	0.01	0.00	0.60	0.00	0.01	0.00	0.55	0.00
	Angular G.	0.03	0.00	0.96	0.00	0.01	0.00	0.95	0.00
	Precuneous Cortex	0.31	0.00	0.85	0.00	0.22	0.00	0.73	0.00
	Parietal Operculum Cortex	0.09	0.00	0.91	0.00	0.27	0.00	0.35	0.00
Temporal	Temporal Pole	0.36	0.77	0.30	0.62	0.22	0.07	0.43	0.16
	Sup. Temporal G., ant. div.	0.69	0.74	0.71	0.94	0.35	0.02	0.50	0.09
	Sup. Temporal G., post. div.	0.49	0.00	0.98	0.01	0.04	0.00	0.44	0.00
	Middle Temporal G., ant. div.	0.76	0.81	0.11	0.43	0.89	0.59	0.87	0.95
	Middle Temporal G., post. div.	0.40	0.05	0.84	0.18	0.59	0.04	0.94	0.17
	Middle Temporal G., temporooccip.	0.33	0.00	0.98	0.00	0.12	0.00	0.40	0.00
	Inf. Temporal G., ant. div.	0.16	0.31	0.42	0.34	0.65	0.77	0.63	0.88
	Inf. Temporal G., post. div.	0.95	0.29	0.13	0.29	0.83	0.01	0.43	0.05
	Inf. Temporal G., temporooccip.	0.78	0.00	0.54	0.02	0.45	0.21	0.09	0.21
	Parahippocampal G., ant. div.	0.57	1.00	0.44	0.84	0.63	0.47	0.16	0.48
	Parahippocampal G., post. div.	0.02	0.18	0.36	0.06	0.12	0.40	0.89	0.36
	Temporal Fusiform Cortex, ant. div.	0.76	0.95	0.02	0.12	0.73	0.61	0.16	0.53
	Temporal Fusiform Cortex, post. div.	0.06	0.63	0.91	0.26	0.25	0.93	0.91	0.72
	Temporal Occipital Fusiform Cortex	0.02	0.63	0.49	0.09	0.03	0.18	0.56	0.09
	Planum Polare	0.70	0.64	0.51	0.85	0.50	0.00	0.85	0.03
	Heschl's G. (includes H1 and H2)	0.03	0.01	0.99	0.01	0.05	0.00	0.46	0.00
	Planum Temporale	0.25	0.00	1.00	0.00	0.03	0.00	0.48	0.00
Occipital	Lateral Occipital Cortex, sup. div.	0.01	0.00	0.67	0.00	0.02	0.00	0.85	0.00
	Lateral Occipital Cortex, inf. div.	0.39	0.00	0.16	0.00	0.13	0.01	0.31	0.02
	Intracalcarine Cortex	0.28	0.00	0.46	0.01	0.13	0.00	0.62	0.00
	Cingulate G., post. div.	0.54	0.00	0.24	0.00	0.38	0.00	0.35	0.00
	Cuneal Cortex	0.03	0.01	0.57	0.01	0.20	0.03	0.84	0.08
	Lingual G.	0.04	0.01	0.57	0.01	0.04	0.09	0.49	0.06
	Occipital Fusiform G.	0.07	0.09	0.80	0.10	0.04	0.93	0.95	0.21
	Supracalcarine Cortex	0.25	0.01	0.45	0.02	0.03	0.00	0.70	0.00
	Occipital Pole	0.32	0.00	0.73	0.01	0.98	0.00	0.35	0.02

Uncorrected p-values of each coefficient shown. Shaded cells indicate values that were significant following FDR correction for multiple comparisons. Tests of each coefficient treated as a statistical family, combining across lobes and hemispheres, for FDR. Abbreviations: div.=division, G.=Gyrus, Suppl.=Supplementary, temporooccip.=temporooccipital, ant.=anterior, post.=posterior, inf.=inferior, sup.=superior.

Supplementary Figure 2. Group differences in MD. Group differences and 95% confidence intervals are shown for each region examined. Positive values indicate ASD>TD. Abbreviations as listed in Supplementary Table 1.

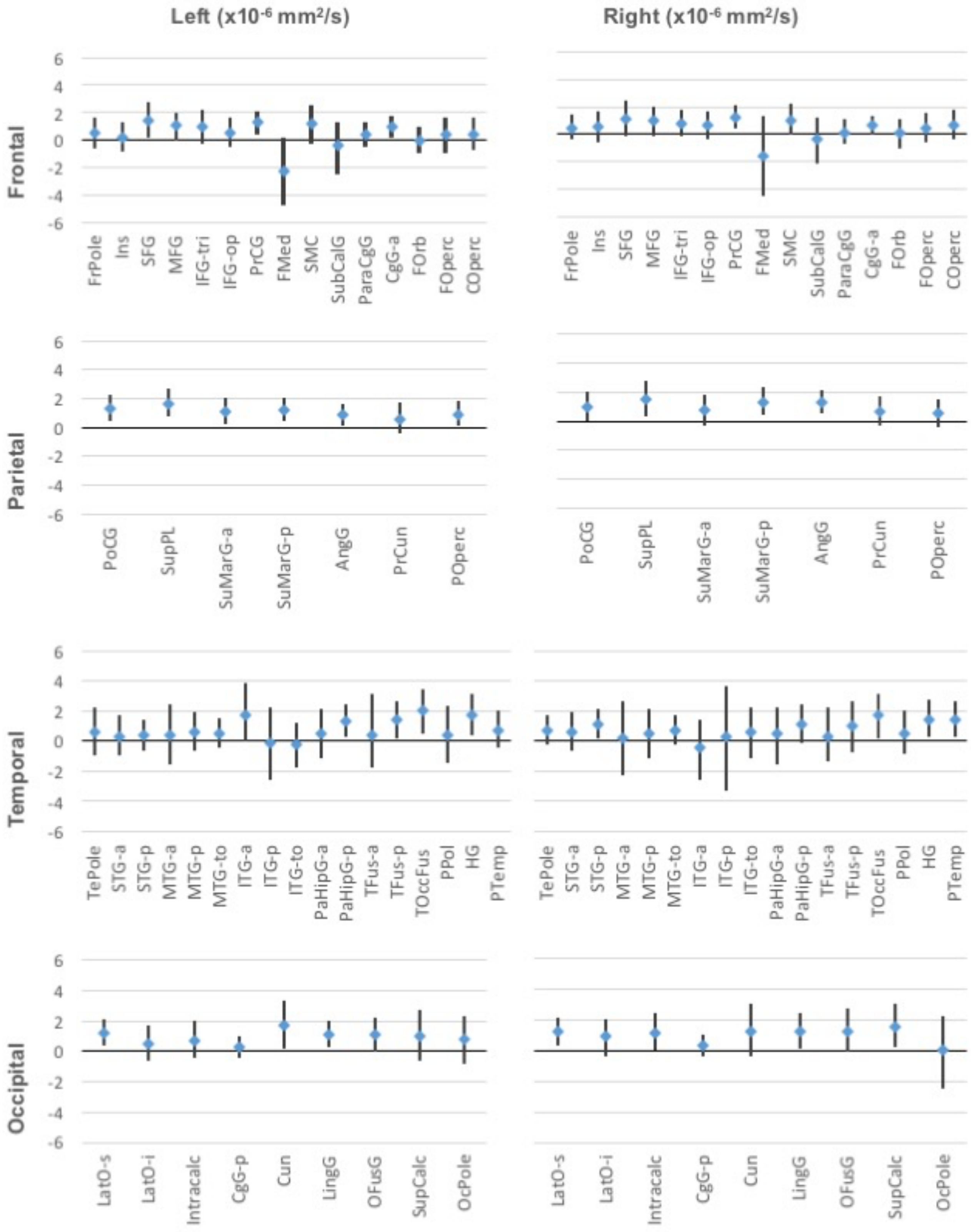

Supplement: Supplementary file 1 [file DataSheet1.pdf]
